# Noncoding RNAs regulate NF-κB signaling to modulate blood vessel inflammation

**DOI:** 10.3389/fgene.2014.00422

**Published:** 2014-12-10

**Authors:** Henry S. Cheng, Makon-Sébastien Njock, Nadiya Khyzha, Lan T. Dang, Jason E. Fish

**Affiliations:** ^1^Toronto General Research Institute, University Health NetworkToronto, ON, Canada; ^2^Department of Laboratory Medicine and Pathobiology, University of TorontoToronto, ON, Canada; ^3^Heart and Stroke/Richard Lewar Centre of Excellence in Cardiovascular ResearchToronto, ON, Canada

**Keywords:** inflammation, atherosclerosis, noncoding RNA, NF-κB, post-transcriptional, signaling, gene regulation, endothelial activation

## Abstract

Cardiovascular diseases such as atherosclerosis are one of the leading causes of morbidity and mortality worldwide. The clinical manifestations of atherosclerosis, which include heart attack and stroke, occur several decades after initiation of the disease and become more severe with age. Inflammation of blood vessels plays a prominent role in atherogenesis. Activation of the endothelium by inflammatory mediators leads to the recruitment of circulating inflammatory cells, which drives atherosclerotic plaque formation and progression. Inflammatory signaling within the endothelium is driven predominantly by the pro-inflammatory transcription factor, NF-κB. Interestingly, activation of NF-κB is enhanced during the normal aging process and this may contribute to the development of cardiovascular disease. Importantly, studies utilizing mouse models of vascular inflammation and atherosclerosis are uncovering a network of noncoding RNAs, particularly microRNAs, which impinge on the NF-κB signaling pathway. Here we summarize the literature regarding the control of vascular inflammation by microRNAs, and provide insight into how these microRNA-based pathways might be harnessed for therapeutic treatment of disease. We also discuss emerging areas of endothelial cell biology, including the involvement of long noncoding RNAs and circulating microRNAs in the control of vascular inflammation.

## AGING, NF-κB ACTIVITY, AND INFLAMMATION

**FIGURE 1:**
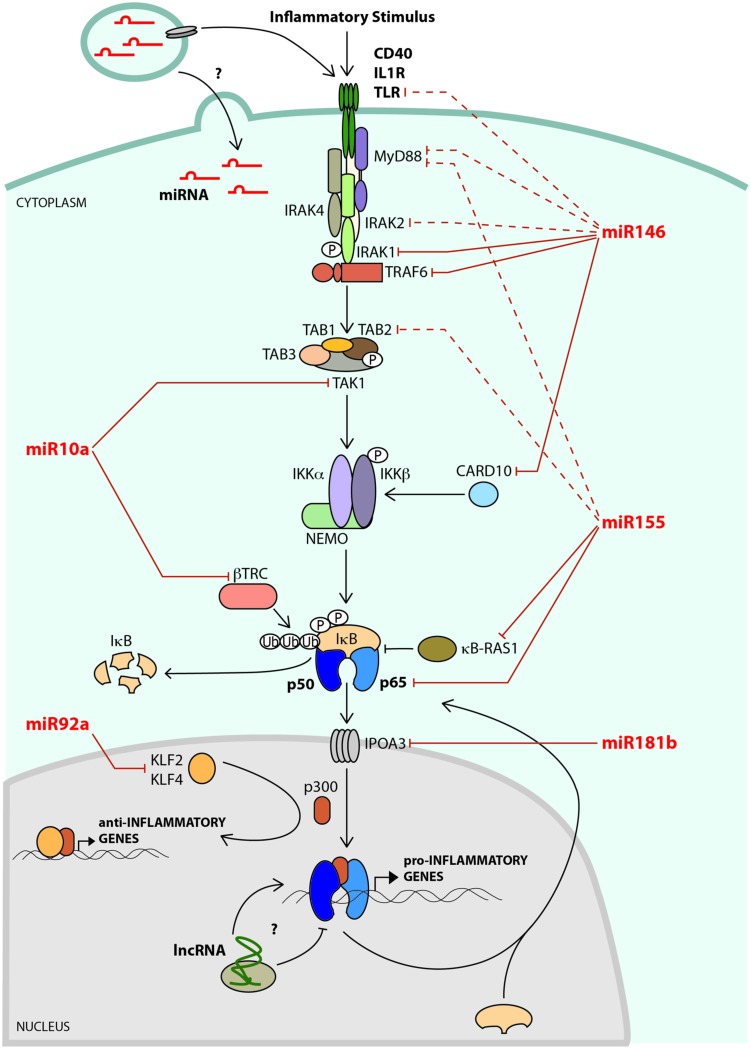
**A network of noncoding RNAs negatively regulates NF-κB signaling.** A key mediator of endothelial cell (EC) activation and vascular inflammation is the transcription factor, NF-κB. In non-stimulated cells, NF-κB subunits (e.g., p65/p50) are sequestered in the cytoplasm through their interaction with IκB. In response to inflammatory signaling, the IKK complex phosphorylates IκB, which is then ubiquitylated by β-TRC, leading to its degradation. This results in the release of NF-κB, allowing it to enter the nucleus and bind to its transcriptional targets, which include leukocyte adhesion molecules, chemokines, and cytokines. NF-κB-dependent microRNAs, such as miR-146a and miR-155 impinge on various stages of the NF-κB signaling pathway, and play critical roles in attenuating activation of this pathway. The microRNA targets that have been verified in ECs are shown as solid lines, and targets validated in other cell types are indicated with dashed lines. Flow dynamics play a crucial role in regulating EC activation. Laminar flow initiates an anti-inflammatory gene expression program that includes up-regulation of transcription factors such as KLF2 and KLF4 that not only promote the expression of anti-inflammatory genes, but also compete with NF-κB for access to the transcriptional co-activator, p300. Laminar flow promotes the expression of miR-10a, which negatively regulates NF-κB activity in ECs by directly targeting *TAK1* and β*-TRC*. In contrast, oscillatory flow induces - while laminar flow suppresses - the expression of miR-92a, which targets KLF2 and KLF4, leading to increased inflammation. MicroRNAs can be found at high levels within microvesicles (MVs) in the circulation. The effects of MVs on EC inflammatory pathways are not known, but may include regulation of signaling pathways or transfer of microRNAs to ECs. Long noncoding RNAs (lncRNA) have recently been shown to regulate NF-κB signaling in other cells types, but their effects on EC inflammatory pathways are not yet known.

While classical risk factors for the development of cardiovascular disease are well established (such as hyperlipidemia, hypertension, smoking, obesity, physical inactivity, and diabetes), age is also a major risk factor, and the incidence and severity of the clinical manifestations of cardiovascular disease rise precipitously with advanced age (Lakatta and Levy, 2003). This is due, in part, to the acquisition of endothelial dysfunction in aging vessels, which reduces vascular responsiveness and contributes to the development of hypertension (Lakatta and Levy, 2003). A key mediator of endothelial dysfunction is the pro-inflammatory transcription factor nuclear factor of κ light polypeptide gene enhancer in B cells (NF-κB; Csiszar et al., 2008). The NF-κB transcription factor is composed of homo- or hetero-dimers of RelA (p65), RelB, c-Rel, p50/p105 (NF-κB1), or p52/p100 (NF-κB2; Hayden and Ghosh, 2004), with p65/p50 heterodimers being the predominant activator of transcription in endothelial cells (ECs). NF-κB is sequestered in the cytoplasm through its interaction with Inhibitor of κB (IκB) under basal conditions. In response to inflammatory signaling, activated IκB kinase (IKK) complex can phosphorylate IκB promoting its proteasomal degradation and thus freeing NF-κB (which contains a nuclear localization signal) to enter the nucleus and bind to its transcriptional targets, which include pro-survival genes, and genes encoding pro-inflammatory cytokines and chemokines and leukocyte adhesion molecules, such as vascular cell adhesion molecule-1 (VCAM-1) and E-selectin (**Figure 1**). Because of the potent inflammatory gene network that is activated by NF-κB, this transcriptional pathway is tightly regulated, and a host of regulatory networks converge on this pathway to inhibit basal NF-κB activity and to resolve NF-κB-mediated inflammatory responses (discussed in more detail below).

Despite the presence of multiple checks and balances that control NF-κB activation, aging leads to chronic low-grade inflammation driven by constitutively elevated levels of nuclear localized, active NF-κB (Helenius et al., 2001). This may be mediated in part by the presence of age-related oxidative stress driven by elevated levels of reactive oxygen species, which can activate NF-κB signaling in the endothelium and promote chronic vascular inflammation (Csiszar et al., 2008). Furthermore, DNA damage accumulated during the aging process can activate NF-κB (Salminen et al., 2012), and circulating levels of the pro-inflammatory cytokine tumor necrosis factor α (TNF-α) also increase with age (Schulz et al., 2004), and this cytokine can activate NF-κB signaling in ECs. Aging also induces a senescence phenotype in ECs, which arrests the proliferation of damaged cells. This cellular state, known as the senescence-associated secretory phenotype (SASP), is associated with constitutively active NF-κB signaling and the secretion of pro-inflammatory cytokines (Salminen et al., 2012). Interestingly, an unbiased assessment of the transcription factor binding motifs that are activated in aging tissue revealed the NF-κB motif as being over-represented, and short-term inhibition of NF-κB signaling could reprogram the gene expression network towards that of young tissue (Adler et al., 2007). Several recent studies have additionally shown that antagonizing NF-κB signaling can delay aging (Osorio et al., 2012; Tilstra et al., 2012), demonstrating the key role that this signaling pathway plays in the aging process of various tissues.

## ATHEROSCLEROSIS IS AN INFLAMMATORY DISEASE OF BLOOD VESSELS

Inflammation plays a prominent role in the pathogenesis of atherosclerosis, a disease characterized by the narrowing of blood vessels due to the growth of an atherosclerotic plaque (Hansson, 2005; Weber and Noels, 2011). In advanced stages of the disease, plaque rupture with associated thrombosis and vessel occlusion can result in myocardial infarction and stroke, which significantly contribute to morbidity and mortality in industrialized countries. During the early stages of atherogenesis, sub-endothelial accumulation of low density lipoprotein (LDL) and EC dysfunction lead to recruitment of circulating monocytes (particularly pro-inflammatory Ly6C^hi^ monocytes; Swirski et al., 2007), which differentiate into macrophages and foam cells within the blood vessel wall (Pober and Sessa, 2007). In response to pro-inflammatory cytokines secreted by these recruited inflammatory cells, ECs become further activated and express high levels of leukocyte adhesion molecules on their cell-surface, which facilitates further monocyte recruitment (Sprague and Khalil, 2009). Because of this reiterative inflammatory process, atherosclerosis is a chronic inflammatory disease that fails to resolve (Hansson, 2005).

Several EC and monocyte/macrophage signaling pathways have been implicated in the pathogenesis of atherosclerosis in mouse models (Weber and Noels, 2011). These include Toll-like receptor 4 (TLR4; Michelsen et al., 2004; den Dekker et al., 2010), CD40 (Donners et al., 2008), and IL-1 receptor (IL-1R) pathways (Devlin et al., 2002; Kirii et al., 2003), among others. These signaling pathways share common adaptor proteins [e.g., TNF receptor-associated factor 6 (TRAF6) and IL-1 receptor-associated kinase 1 (IRAK1); Cao et al., 1996; Lomaga et al., 1999; Hull et al., 2002; Donners et al., 2008], and signaling through these adaptors results in the induction of transcription factors of the NF-κB (**Figure 1**), activator protein-1 (AP-1) and early growth response (EGR) families. Upon activation in ECs, these transcription factors cooperatively drive the transcription of inducible adhesion molecules [e.g., *VCAM-1, inducible cell adhesion molecule-1* (*ICAM-1*), E-selectin (*SELE*)], cytokines [e.g., *TNF-*α, *Interleukin-1*β (*IL-1*β)] and chemokines [e.g., *monocyte chemoattractant protein-1* (*MCP-1*)] (Ahmad et al., 1998; Wieland et al., 2005). Importantly, antagonizing NF-κB (Gareus et al., 2008), AP-1 (Wang et al., 2011a), or EGR (Harja et al., 2004; Albrecht et al., 2010) pathways inhibits atherogenesis. It is important to note that while inhibition of NF-κB activity in the endothelium reduces EC activation and atherogenesis (Gareus et al., 2008), antagonizing NF-κB in macrophages worsens atherosclerosis (Kanters et al., 2003), underscoring the diversity of NF-κB function in distinct tissues that are involved in atherogenesis.

## NEGATIVE FEEDBACK LOOPS ACT TO RESTRAIN THE INTENSITY AND DURATION OF NF-κB ACTIVITY

Inflammation proceeds through a series of well-orchestrated steps, ending in resolution (Liew et al., 2005; Serhan et al., 2007). A defect in resolution appears to contribute to atherogenesis since chronic vascular inflammation is a hallmark of this disease (Hansson, 2005; Weber and Noels, 2011). Importantly, inflammatory signals also induce the expression of proteins (Serhan et al., 2007), microRNAs, and lncRNAs (discussed in detail below) that mediate resolution at a later stage of inflammation: thereby forming negative feedback loops. In the context of vascular inflammation, transcription of adhesion molecule genes in ECs must be limited to prevent continual recruitment of leukocytes (Winsauer and de Martin, 2007). A classic example of a negative feedback loop is the transcriptional induction of IκB by NF-κB. IκB facilitates the transport of NF-κB out of the nucleus to attenuate NF-κB-dependent transcription (Arenzana-Seisdedos et al., 1995; **Figure 1**). Several other negative regulatory loops have also been well characterized. For example, IRAK-M is induced by inflammatory stimuli and is a kinase-deficient homolog of IRAK1 that inhibits signaling through IRAK1 (Wesche et al., 1999; Kobayashi et al., 2002), and MyD88s, an alternatively spliced product of MyD88, is also induced and antagonizes MyD88 to inhibit signaling (Janssens et al., 2002, 2003). Additionally, A20 (also known as *TNFAIP3*) is induced by NF-κB and negatively regulates inflammatory signaling by removing activating (i.e., Lys^63^) ubiquitin marks on adaptor molecules such as RIP1 and TRAF6 (Boone et al., 2004; Wertz et al., 2004). Deletion of A20 impairs the resolution of inflammation (Boone et al., 2004; Turer et al., 2008) and enhances atherogenesis (Wolfrum et al., 2007). IRAK1 protein is also degraded by the proteasome following signaling, which serves to attenuate signal transduction (Yamin and Miller, 1997).

## NONCODING RNAs IN GENE REGULATION

In addition to the identification of proteins that serve as negative feedback regulators of NF-κB, noncoding RNAs, including microRNAs (discussed below) and long noncoding RNAs (lncRNAs; discussed later), have been shown to regulate inflammatory signaling. MicroRNAs are short RNAs (∼21–23 nts) that mediate post-transcriptional gene regulation (Bartel, 2009). They are transcribed by RNA Polymerase II (Lee et al., 2004), and following export/processing into the mature microRNA (Grishok et al., 2001; Lee et al., 2003; Lund et al., 2004), one strand of the double-stranded microRNA (the guide strand) is incorporated into the RNA-induced silencing complex (RISC), which binds to target messenger RNAs (mRNAs) through partial base-pair complementarity (Gregory et al., 2005). In general, microRNAs bind to the 3′ untranslated regions (UTRs) of target mRNAs and negatively regulate mRNA stability and translation (Baek et al., 2008). Experimental validation has demonstrated that a single microRNA can directly repress the expression of 100s of mRNAs (Baek et al., 2008). However, only a few microRNA targets may be critical in dictating the cellular function of a microRNA (Valastyan et al., 2009). Importantly, mice deficient in just a single microRNA have profound functional deficits in specific cellular pathways (Thai et al., 2007; van Rooij et al., 2007; Wang et al., 2008; Boldin et al., 2011), suggesting that microRNAs potently regulate key cellular activities.

The central role of NF-κB in driving inflammation necessitates tight control of this pathway. A growing number of microRNAs have been implicated in the regulation of NF-κB signaling (Boldin and Baltimore, 2012; Gantier et al., 2012; Song et al., 2013). For the purposes of this review, we will focus on microRNAs that influence this pathway in ECs. For example, miR-146a is induced in ECs by inflammatory stimuli and acts to terminate signaling by targeting upstream components of the NF-κB signaling pathway (Cheng et al., 2013). Other microRNAs, such as miR-10a (Fang et al., 2010) and miR-92a (Wu et al., 2011; Fang and Davies, 2012; Loyer et al., 2014), are regulated by laminar flow, a potent inhibitor of EC inflammatory pathways. In addition, miR-181b is down-regulated in vascular inflammatory diseases such as sepsis and coronary artery disease (CAD) and functions as an antagonist of the nuclear import of NF-κB subunits in ECs (Sun et al., 2012, 2014). Although it is not the focus of this review, microRNAs have also been identified that directly target key leukocyte adhesion molecules in activated ECs [e.g., miR-126 targets *VCAM1* (Harris et al., 2008), miR-31 targets *SELE* and miR-17-3p targets *ICAM1* (Suarez et al., 2010)].

## MicroRNA-146a (miR-146a) PARTICIPATES IN NEGATIVE FEEDBACK REGULATION OF NF-κB ACTIVITY AND CONTRIBUTES TO THE CONTROL OF VASCULAR INFLAMMATION

Several microRNAs are induced in response to inflammatory stimuli in human monocytes (Taganov et al., 2006; Bazzoni et al., 2009) and ECs (Suarez et al., 2010; Cheng et al., 2013). Interestingly, the NF-κB-dependent induction of *miR-146a* plays a critical role in attenuating NF-κB signaling. This is accomplished through the targeting of TRAF6 and IRAK1, two adaptor proteins that act upstream of the NF-κB pathway (Taganov et al., 2006; Cheng et al., 2013; **Figure 1**). Interestingly, we found that while the transcription of *miR-146a* occurs very early during the inflammatory response in ECs, mature miR-146a does not accumulate until late in the response, and miR-146a accumulation coincides with the resolution of the inflammatory response (Cheng et al., 2013). Cultured human ECs treated with miR-146 inhibitors, as well as *miR-146a^-/-^* mice, have an enhanced magnitude and duration of the EC inflammatory response; thus indicating the functional importance of this microRNA in repressing vascular inflammation (Cheng et al., 2013). In contrast, over-expression of miR-146a in human ECs suppresses their activation and inhibits monocyte adhesion (Cheng et al., 2013). In addition to the targeting of IRAK1/TRAF6, we also identified the RNA binding protein, HuR, as a novel miR-146a target, and found that HuR promotes EC activation through the repression of an anti-inflammatory Kruppel-like Factor 2 (KLF2)/eNOS pathway (Cheng et al., 2013). More recently, the caspase recruitment domain family 10 (CARD10), an adaptor protein for GPCR-mediated NF-κB activity, was identified as a target of miR-146a in cultured human ECs (Cowan et al., 2014; Rau et al., 2014). MiR-146a/b were also previously found to be highly elevated in senescent human fibroblasts (Bhaumik et al., 2009) and ECs (Vasa-Nicotera et al., 2011; Olivieri et al., 2013), and ectopic expression of miR-146a suppressed the SASP phenotype of senescent cells (Bhaumik et al., 2009).

In addition to the anti-inflammatory role of miR-146a in ECs, this microRNA also plays several important roles in repressing inflammatory signaling in immune cells. In monocytes for example, miR-146a participates in endotoxin tolerance elicited by lipopolysaccharide (LPS), a bacterial cell wall component. Following initial exposure to a low dose of LPS, miR-146a expression is induced and maintained, allowing for suppression of a subsequent inflammatory response to a high dose of LPS (Biswas and Lopez-Collazo, 2009). Antagonism of miR-146a induction in human cultured monocytes and in mouse models prevents endotoxin tolerance from occurring (Nahid et al., 2009; Banerjee et al., 2013) and *miR-146a^-/-^* mice are hypersensitive to LPS, and produce extremely high levels of pro-inflammatory cytokines that cause lethal septic shock (Boldin et al., 2011). The expression of miR-146a is also down-regulated in macrophages exposed to oxidized LDL. Over-expression of miR-146a inhibits LDL cholesterol uptake by macrophages and the secretion of pro-inflammatory cytokines through targeting of TLR4 (Yang et al., 2011). Furthermore, *miR-146a^-/-^* mice produce an expanded population of pro-inflammatory Ly6C^hi^ monocytes in response to inflammatory stimulation (Etzrodt et al., 2012), suggesting that the innate inflammatory response may be exaggerated and prolonged in these mice. These mice also have protracted T-cell responses (Yang et al., 2012), defective regulatory T-cell functions (Lu et al., 2010), and develop an autoimmune-like myeloproliferative disease later in life (Zhao et al., 2011), suggesting that miR-146a-mediated feedback loops are necessary to prevent prolonged activation of the immune system.

The role of miR-146a in atherosclerosis remains to be tested. The collective data above support an anti-inflammatory and anti-atherosclerotic role for miR-146a in ECs and leukocytes. Interestingly, circulating levels of miR-146a increase during atherogenesis in mice (Sun et al., 2014), and elevated expression of miR-146a is observed in atherosclerotic plaques in mice (Nazari-Jahantigh et al., 2012) and in humans (Raitoharju et al., 2011). This increase in miR-146a expression may be due to the activation of inflammatory pathways during the course of disease progression. Deletion of *miR-146a* or delivery of miR-146a mimics will be informative to define the role of this microRNA in atherogenesis.

## MiR-10a AND miR-92a CONTRIBUTE TO THE REGULATION OF NF-κB IN RESPONSE TO BLOOD FLOW

Flow dynamics play a crucial role in regulating blood vessel biology, and regional differences in these dynamics affect inflammation and the progression of atherosclerosis. Areas of the vasculature that are exposed to uniform laminar flow are protected against inflammation and the development of atherosclerosis, while regions of disturbed flow, which are typically found at branch points, bifurcations, and the lesser (inner) curvature of arched vessels, are prone to inflammatory activation and plaque formation (Dai et al., 2004; VanderLaan et al., 2004). Laminar flow initiates a gene expression program that includes up-regulation of atheroprotective transcription factors such as KLF2 (SenBanerjee et al., 2004), and inhibition of pro-inflammatory transcription factors such as NF-κB (Dai et al., 2004; Won et al., 2007). Further to transcriptional programs, blood flow also modulates the expression of several microRNAs (Qin et al., 2010; Wang et al., 2010b; Weber et al., 2010b). To identify microRNAs that might contribute to the regulation of vascular inflammation, Fang et al. (2010) performed microRNA arrays on atherosusceptible versus atheroprotective regions of the vasculature in swine models, and found that miR-10a is an EC-enriched microRNA that is decreased in regions that are prone to the development of atherosclerosis, such as the lesser curvature of the aortic arch. The differential flow-mediated regulation of miR-10a in the vasculature was confirmed in mouse models (Fang et al., 2010). These results suggested that laminar flow promotes the expression of miR-10a; however, it should be noted that miR-10a does not appear to be regulated by KLF2 (Hergenreider et al., 2012). The aortic arch experiences disturbed flow dynamics and elevated NF-κB activity, which suggests that NF-κB may negatively regulate miR-10a expression. In support of this, Xue et al. (2011) showed that miR-10a is downregulated by TLR-mediated NF-κB activity in intestinal dendritic cells. Elucidating the mechanisms responsible for miR-10a flow-dependent regulation *in vivo* will require further investigation. Functional characterization of this microRNA revealed that miR-10a negatively regulates NF-κB activity in cultured human ECs by directly targeting *MAP3K7* (also known as TAK1) and β*-TRC* (Fang et al., 2010; **Figure 1**). TAK1 is essential for NF-κB signaling (Sato et al., 2005) as it is a kinase that activates IKKβ, which is responsible for IκBα phosphorylation, while β-TRC mediates ubiquitination of phosphorylated IκBα, facilitating ubiquitination-mediated protein degradation (Yaron et al., 1998). The role of miR-10a in atherosclerosis has not been tested, but the results of Fang et al. (2010) suggest that miR-10a may suppress atherogenesis; linking flow dynamics with NF-κB signaling. The recent generation of *miR-10a* knock-out mice (Stadthagen et al., 2013) will be useful to test this hypothesis.

A distinct set of microRNAs is induced by disturbed or oscillatory flow in cultured human cells. For example, miR-663 is upregulated by oscillatory flow and drives a pro-inflammatory expression profile and enhances monocyte adhesion to the endothelium (Ni et al., 2011). Oscillatory flow also induces the expression of miR-92a in human cells, while atheroprotective laminar flow down-regulates its expression (Wu et al., 2011). Interestingly, *KLF2* and *KLF4* have been shown to be miR-92a target genes (Wu et al., 2011; Fang and Davies, 2012). These two transcription factors inhibit NF-κB dependent inflammatory genes (SenBanerjee et al., 2004; Hamik et al., 2007) in part by competing with NF-κB for access to the transcriptional co-activators p300/CBP (SenBanerjee et al., 2004; Zhou et al., 2012) (**Figure 1**). In mice, genetic deficiency of either *KLF2* or *KLF4* in *ApoE*^-/-^ mice (which develop atherosclerotic lesions when placed on high fat diet) enhances atherosclerosis, indicating an atheroprotective role for KLF2 and KLF4 (Atkins et al., 2008; Zhou et al., 2012). Furthermore, a role for miR-92a-dependent regulation of KLF2/KLF4 in the pathogenesis of atherosclerosis was recently demonstrated. Loyer et al. (2014) found that endothelial miR-92a expression is induced by a combination of low shear stress and oxidized LDL, two key factors that drive EC activation, and they observed that miR-92a levels are enhanced during atherogenesis in mouse models. Using miR-92a inhibitors, they observed an increase in KLF2 and KLF4 levels as well as a decrease in total and phosphorylated p65 in the aortas of atherosclerotic mice, which was accompanied by diminished atherosclerotic plaque formation (Loyer et al., 2014). Thus, miR-92a appears to enhance NF-κB signaling at two levels: by repressing KLF2/KLF4, antagonists of NF-κB-dependent transcription, and by promoting the activation of p65. The mechanisms responsible for this latter effect on p65 are not known. Collectively, these studies underscore the pro-inflammatory and pro-atherogenic function of miR-92a in the endothelium, and link this microRNA with regulation of flow-dependent transcriptional programs.

## MiR-181b IS DOWN-REGULATED IN VASCULAR INFLAMMATORY DISEASES AND CONTROLS THE IMPORT OF NF-κB INTO THE NUCLEUS

To identify microRNAs that might be involved in the inflammatory response, Sun et al. (2012) profiled microRNA expression in human ECs exposed to the pro-inflammatory cytokine, TNF-α. They found that miR-181b was rapidly down-regulated by this stimulus. The miR-181 family consists of four members (*miR-181a, b, c*, and *d*) in human and mouse. The predominant isoform in ECs is miR-181b, which is expressed at greater than 10-fold higher levels than miR-181a, while the other two isoforms are nearly undetectable (Sun et al., 2012). Importantly, circulating levels of miR-181b are decreased in patients with sepsis, a systemic inflammatory response that is associated with EC activation, vascular permeability, and severe organ damage (Sun et al., 2012). This microRNA is also down-regulated in the circulation and in the intima of atherosclerotic lesions in mouse models of atherosclerosis, and circulating levels are lower in patients with CAD (Sun et al., 2014). This suggests that down-regulation of miR-181b occurs in diverse vascular inflammatory conditions. The over-expression of miR-181b in cultured human ECs or systemic delivery of miR-181b mimics in mice represses NF-κB dependent vascular inflammatory gene expression. Treatment with miR-181b mimics also decreases leukocyte recruitment and damage to the lung, and increases survivability in a mouse model of sepsis (Sun et al., 2012). Systemic mimic injections resulted in miR-181b accumulation in the intimal region (i.e., ECs) of the aorta and in circulating leukocytes, with limited accumulation in the medial layer of the vessel wall. With success in systemic delivery of miR-181b mimic into mice in an acute inflammatory condition (i.e., sepsis), their subsequent study demonstrated that multiple injections of miR-181b mimic can reduce vascular inflammation and reduce lipid-rich plaque accumulation in mouse models of atherosclerosis (Sun et al., 2014).

By analyzing the targets of miR-181b, Sun et al. (2012) found that this microRNA impinges on the NF-κB pathway by targeting the nuclear protein transporter IPOA3 (*Importin-3α*) in human and mouse ECs (**Figure 1**). The IPOA family has been shown to mediate nuclear import of NF-κB subunits during the inflammatory response (Fagerlund et al., 2005). Interestingly, the miR-181b-mediated repression of NF-κB activity was only observed in the endothelium and not in leukocytes, despite efficient delivery of miR-181b to leukocytes (Sun et al., 2014). While miR-181b represses IPOA3 expression in leukocytes, the main isoform used for NF-κB nuclear transport in leukocytes is IPOA5 (which is not targeted by miR-181b): explaining the insensitivity of leukocytes to miR-181b manipulation. This is an important finding considering that inhibition of NF-κB in ECs and leukocytes can have opposite effects on atherogenesis (Kanters et al., 2003; Gareus et al., 2008). Collectively, these studies highlight the importance, and potential therapeutic relevance, of miR-181b in vascular inflammatory diseases.

## MiR-155 HAS PLEIOTROPIC ROLES IN CONTROLLING INFLAMMATION

Since microRNAs can target and repress several genes, they can have complex effects on signaling pathways. MiR-155 has been intensely studied for its role in controlling inflammation, but in contrast to miR-146a, miR-10a, miR-92a, and miR-181b, which appear to have predominantly pro- or anti-inflammatory roles, studies on miR-155 have often revealed conflicting roles for this microRNA. Many of these differences seem to be attributable to the cell type being studied. For example, *miR-155^-/-^* mice are severely immunocompromised (Rodriguez et al., 2007), and this appears to be dependent on miR-155 function in B-cells (Vigorito et al., 2007). These mice are also resistant to auto-immunity through T-cell mediated effects of miR-155 (O’Connell et al., 2010). A role for miR-155 in leukocytes during atherogenesis has also been demonstrated. The levels of miR-155 dramatically increase in atherosclerotic plaques and within plaque macrophages in mice (Nazari-Jahantigh et al., 2012; Tian et al., 2014). By utilizing bone marrow transplant approaches, one report has found that miR-155 promotes the development of atherosclerotic plaques in the *ApoE^-/-^* model by driving an NF-κB-dependent pro-inflammatory response (Nazari-Jahantigh et al., 2012), whereas another group has found that miR-155 inhibits atherosclerosis in the *Ldlr-/-* model by antagonizing the levels of circulating neutrophils and pro-inflammatory Ly6C^hi^ monocytes (Donners et al., 2012). In addition, injection of miR-155 inhibitors has been shown to reduce plaque formation in *ApoE^-/-^* mice, and this is accompanied by reduced ox-LDL uptake and less reactive oxygen species production (Tian et al., 2014). Additional investigations will be required to resolve the differences in these studies, which used different atherosclerotic mouse models and assessed different time-points of disease progression.

Several studies have assessed miR-155 function in vascular ECs and have found a largely anti-inflammatory effect. However, it is important to note that only *in vitro* experiments have been performed thus far. For example, miR-155 can target angiotensin II type I receptor (AGTR1) and ETS1 in human ECs. Angiotensin II (Ang II) is a potent inducer of inflammation, and ETS1 has been shown to drive the expression of VCAM1 and MCP1 in response to Ang II stimulation. Thus, over-expression of miR-155 inhibits the pro-inflammatory effects of Ang II (Zhu et al., 2011). MiR-155 is also induced by the pro-inflammatory cytokine, TNF-α, and can act as a negative feedback regulator by directly targeting p65 and inhibiting human EC activation (Wu et al., 2014b). A recent report elegantly demonstrated that miR-155 expression is repressed by Notch signaling in mouse bone marrow stromal ECs (Wang et al., 2014). Deletion of Notch in these cells enhances miR-155 expression and miR-155 can target the NF-κB inhibitor, κB-Ras1, enhancing NF-κB activity in stromal ECs and driving pro-inflammatory cytokine production and myeloproliferation. MiR-155 has also been shown to antagonize NF-κB signaling in other cell types. For example, miR-155 can target MyD88 in human macrophages (Huang et al., 2010), and TAB2 in human dendritic cells (Ceppi et al., 2009). MiR-155 also negatively regulates NF-κB signaling in human epithelial cells during *H. pylori* infection (Xiao et al., 2009). Taken together, the role of miR-155 in controlling vascular inflammation appears to be highly complex and cell-specific, and further investigation is required to fully understand the role of this microRNA in vascular pathology.

## LncRNAs ARE AN INTEGRAL PART OF THE NF-κB SIGNALING NETWORK, BUT THEY HAVE AN UNEXPLORED ROLE IN VASCULAR INFLAMMATION

Over the past few years, lncRNA have gained increasing recognition as regulatory molecules. By definition, lncRNAs are classified as RNA transcripts greater than 200 nucleotides in length that do not code for a functional protein (Sabin et al., 2013). Similarly to mRNAs, lncRNAs are RNA polymerase II transcribed, can be polyadenylated at their 3′ end, and are often spliced. However, lncRNAs differ from mRNAs in their low conservation across species, low expression levels, and in many cases, nuclear accumulation (Guil and Esteller, 2012). LncRNAs are located in various genomic regions; for example, they can be expressed as intergenic genes, they can be found within introns of existing protein-coding genes, they can be antisense to protein-coding genes, or they can be transcribed from enhancer regions (Schonrock et al., 2012). Although lncRNA functions are highly diverse and are continuing to be uncovered, the most frequently reported function is their involvement in chromatin remodeling. In fact, Guttman et al. (2011) showed that 30% of lncRNAs in mouse embryonic stem cells interact with at least one chromatin remodeling complex. Of these, the two most commonly described are polycomb group (PcG) and trithorax group (TxG) complexes, which deposit repressive H3K27me3 or activating H3K4me3 histone marks, respectively. In addition, other lncRNA functions include, but are not limited to: recruitment of transcription factors to chromatin targets, acting as decoys for cellular proteins, affecting mRNA stability, facilitating chromatin looping, interacting with microRNAs to modulate their function or processing, and acting as a scaffold for protein complex formation (Rinn and Chang, 2012; Cech and Steitz, 2014; Yang et al., 2014; Yoon et al., 2014).

While the involvement of lncRNAs in EC biology is just beginning to be explored (Bell et al., 2014; Ge et al., 2014; Michalik et al., 2014), studies in other cell types have uncovered roles for lncRNAs in the control of NF-κB signaling and inflammation. One of the first studies to demonstrate the involvement of lncRNAs in the inflammatory response was the demonstration that a lncRNA located ∼50 kb downstream of mouse *Cox2* (named *lncRNA-Cox2*) is induced together with *Cox2* in mouse bone marrow-derived macrophages upon exposure to pro-inflammatory stimuli such as LPS (Carpenter et al., 2013). Knock-down of *lncRNA-Cox2* and subsequent RNA-seq experiments revealed that *lncRNA-Cox2* is capable of affecting the expression of over a thousand genes, but does not influence the expression of *Cox2* itself (Carpenter et al., 2013). There were 787 up-regulated genes and 713 down-regulated genes with gene ontology enrichment analysis revealing a significant over-representation of immune response genes. Although no explanation for how *lncRNA-Cox2* can induce gene expression was provided, the group did show that *lncRNA-Cox2* can interact with heterogeneous nuclear ribonucleoproteins (hnRNP) A/B and A2/B1 to down-regulate target genes (Carpenter et al., 2013). HnRNPs are nuclear complexes that affect mRNA processing and stability (Krecic and Swanson, 1999). Thus, this lncRNA–hnRNP interaction provides a model for how this lncRNA can act in *trans* (i.e., on distal loci) to concurrently affect the expression of a large number of genes (Carpenter et al., 2013). While this lncRNA has not been shown to directly influence NF-κB signaling, this example illustrates how lncRNAs are integrated into the inflammatory gene regulatory network.

In the human genome, a distinct lncRNA is located in proximity to the *COX2* gene. Characterized by Krawczyk and Emerson (2014), this lncRNA was named P50-associated COX-2 extragenic RNA (*PACER*), is located 1 kb upstream of *COX2*, and is transcribed in the antisense direction. Similar to *lncRNA-Cox2*, *PACER* expression is induced following exposure of human macrophages/monocytes to pro-inflammatory stimuli such as LPS. Under basal conditions *PACER* expression is repressed, but LPS stimulation leads to recruitment of CTCF/cohesin and establishment of an open chromatin environment, allowing for both *PACER* and *COX2* expression. *PACER* transcripts are retained in the nucleus, of which one third are associated with chromatin. Unlike *lncRNA-Cox2*, *PACER* knock-down affects *COX2* expression in *cis* (i.e., within the same locus). The mechanism involves the physical interaction of *PACER* transcripts with the p50 NF-κB subunit. Thus, *PACER* acts to impede p50 homodimers from binding to the *COX2* promoter. Since p50 homodimers are repressive, *PACER* facilitates the binding of activating p65/p50 heterodimers rather than p50/p50 homodimers to the *COX2* promoter, resulting in p300 recruitment, deposition of active histone acetylation marks throughout the locus, and robust *COX2* transcription. Therefore, *PACER* is induced by inflammatory stimuli and acts in *cis* to maintain NF-κB-dependent transcription at the *COX2* locus. Whether *PACER*-p50 interactions affect the expression of other NF-κB-regulated genes in *trans* remains to be investigated.

*PACER* is not the only lncRNA reported to physically interact with NF-κB subunits. A study by Rapicavoli et al. (2013) discovered another lncRNA in mice, *Lethe*, which interacts with the p65 NF-κB subunit. *Lethe* was identified as the lncRNA with the highest fold change upon TNF-α or IL-1β treatment of mouse embryonic fibroblasts. Further characterization revealed that *Lethe* is enriched in the nucleus and is associated with chromatin. Within the nucleus, *Lethe* acts as a negative regulator of NF-κB signaling by binding and sequestering the p65 subunit. Therefore, *Lethe* participates in a negative feedback loop, as it is an NF-κB inducible transcript, which then acts to turn off expression of NF-κB-regulated genes. Whether *Lethe* has a human ortholog or has a function in ECs is unknown.

Recently, noncoding RNAs produced from gene enhancers [e.g., enhancer RNAs (eRNAs) and regions of bidirectional transcription (RBT)] have been shown to play a critical role in gene induction (Lam et al., 2014). The function of eRNAs is still poorly understood, but they appear to promote transcription by mediating gene looping between enhancers and promoters through their recruitment of cohesin complex components (Li et al., 2013b), or by influencing chromatin accessibility of gene promoters (Mousavi et al., 2013). A large number (>100) of eRNAs and RBTs are induced in human monocytes stimulated with LPS (IIott et al., 2014). Within the *IL-1*β locus, one eRNA (*IL-1*β*-eRNA*) located downstream, and two RBTs [*IL-1*β*-RBT-46(+)* and *IL-1*β*-RBT-46(-)*] located upstream of *IL-1*β are induced by LPS stimulation. These transcripts are NF-κB-dependent and are enriched in the nucleus, and their induction kinetics mirror that of *IL-1*β mRNA (IIott et al., 2014). Typical of eRNAs/RBTs, *IL-1*β*-eRNA* and *IL-1*β*-RBT-46* were shown to act in *cis*, as their silencing led to decreased *IL-1*β mRNA and IL-1β protein levels. However, these eRNAs/RBTs may have additional direct and indirect target genes since the expression of *CXCL8* (which is located on a different chromosome), was also affected by their knock-down. Thus, eRNAs are also integrated into the NF-κB signaling network, but their mechanisms of action are poorly understood.

Despite our increasing appreciation of the impact of lncRNAs in the context of the inflammatory response, many questions remain unanswered. In particular, the effect of lncRNAs on NF-κB signaling is only beginning to be studied, with the majority of studies thus far performed in monocytes/macrophages *ex vivo*. The role of lncRNAs in EC inflammatory pathways is currently unknown. Since many lncRNAs are expressed in a cell-type specific fashion, there is likely to be a wealth of novel lncRNAs to be uncovered that may participate in EC inflammation and atherosclerosis. Interestingly, recent studies are beginning to implicate lncRNAs in controlling atherogenesis. For example, *lncRNA-p21* is down-regulated in mouse models of atherosclerosis (Wu et al., 2014a). Further characterization revealed that this lncRNA represses the proliferation of smooth muscle cells and its inhibition in a mouse carotid artery injury model enhances neointimal formation (Wu et al., 2014a). In addition, the lncRNA *ANRIL*, which is located in the 9p21.3 CAD susceptibility locus in humans (McPherson et al., 2007), appears to regulate smooth muscle cell proliferation (Jarinova et al., 2009; Motterle et al., 2012; Holdt et al., 2013), but the mechanism of action is still incompletely understood (Chen et al., 2014). These findings demonstrate that lncRNAs are likely to have a major impact on atherosclerotic disease, providing a strong impetus to further identify the lncRNAs involved in controlling the vascular inflammatory response.

## LEVELS OF CIRCULATING microRNAs ARE ALTERED IN CARDIOVASCULAR DISEASE, BUT THEIR FUNCTIONS ARE POORLY UNDERSTOOD

Recent studies have reported the presence of microRNAs in conditioned media *in vitro* (Zhang et al., 2010), as well as in blood, and other body fluids (e.g., saliva, urine, and milk; Weber et al., 2010a). MicroRNAs can be secreted from cells within small membrane-bound vesicles called microvesicles (MVs), or they can be associated with RNA-binding proteins such as Argonaute 2 (Arroyo et al., 2011) or Nucleophosmin (Wang et al., 2010a). The packaging of microRNAs into MVs or protein complexes protects them from degradation and confers the surprisingly high stability of microRNAs in blood and other body fluids (Mitchell et al., 2008). Three types of MVs are released by cells: microparticles (MPs), exosomes, and apoptotic bodies (ABs; Mittelbrunn and Sanchez-Madrid, 2012). MPs are a heterogeneous population of vesicles (100–1000 nm) produced by plasma membrane blebbing due to the disruption of membrane phospholipid asymmetry (Hamon et al., 2000). These vesicles are released from most cell types in response to stress, such as cell activation or apoptosis (Combes et al., 1999; Simoncini et al., 2009; Leroyer et al., 2010). In contrast, exosomes are smaller and more homogeneous in size (30–100 nm) and are formed as intraluminal vesicles inside multivesicular bodies of the endosomal compartment (Heijnen et al., 1999; Simons and Raposo, 2009). They are released into the extracellular environment by exocytosis in a constitutive manner and in high quantities. ABs, the largest vesicles (1–5 μm), are membrane blebs released from apoptotic cells (Hristov et al., 2004). In addition to microRNAs, MVs also contain various membrane proteins and lipids, soluble proteins and genetic information (i.e., mRNA and DNA) derived from their parental cell. Interestingly, it appears that some microRNAs may be selectively loaded or excluded from MVs, since their abundance differs from their parental cell (Pigati et al., 2010; Li et al., 2013a).

Several studies have reported the presence of MPs in the plasma of healthy volunteers, and have found that MP levels increase dramatically under pathological conditions, such as cardiovascular diseases. For example, the levels of endothelial-derived MPs are elevated in patients with CAD (Mallat et al., 2000; Bernal-Mizrachi et al., 2003; Bulut et al., 2009; Nozaki et al., 2009). Furthermore, high concentrations of leukocyte-derived plasma MPs are associated with unstable plaque in high-grade carotid stenosis patients (Sarlon-Bartoli et al., 2013). Thus, a high level of MPs in the blood is a potential diagnostic biomarker for cardiovascular risk. MPs have potent pro-inflammatory properties, as MPs derived from LPS-stimulated monocytes can activate ECs (Wang et al., 2011b), and conversely, MPs produced by ECs can activate monocytes (Jy et al., 2004). However, whether microRNA transfer is involved in the pro-inflammatory effects of MPs has not yet been studied. Interestingly, microRNA content in the blood can be used as a biomarker to identify patients at risk for cardiovascular diseases. Fichtlscherer et al. (2010) have shown that the circulating levels of vascular microRNAs, including miR-126 and two members of the miR-17-92 cluster (miR-17 and miR-92a) are decreased in CAD patients compared to healthy controls, whereas cardiac muscle microRNAs (miR-133a, miR-208a) are enriched. Inflammation-associated microRNAs are also potential biomarkers for vascular diseases, since miR-155 and miR-181b are reduced in human plasma from patients with CAD (Fichtlscherer et al., 2010; Sun et al., 2014).

Besides their potential for being utilized as diagnostic biomarkers for disease, MVs are capable of modulating numerous (patho-) physiological processes, including thrombosis, inflammation, angiogenesis, and vascular tone (VanWijk et al., 2003; Leroyer et al., 2010). They contain bioactive molecules (i.e., lipids and proteins) that give them an intrinsic biological activity (Simoncini et al., 2009; Leroyer et al., 2010; Lacroix et al., 2012), and they can also participate in intercellular communication by transferring functional mRNAs and microRNAs to recipient cells (Zernecke et al., 2009; Zhang et al., 2010; Hergenreider et al., 2012; Zhou et al., 2013; Bang et al., 2014; Okoye et al., 2014). For example, Hergenreider et al. (2012) showed that miR-143/145 are enriched in MVs secreted from cultured human ECs exposed to laminar flow or from ECs over-expressing the shear-responsive transcription factor KLF2. These endothelial MVs can induce an atheroprotective phenotype in co-cultured smooth muscle cells *in vitro*. Moreover, injection of endothelial-derived miR-143/145-containing MVs reduces the formation of atherosclerotic lesions in the aortas of atherosclerotic mice (Hergenreider et al., 2012). In another study, Zernecke et al. (2009) demonstrated that endothelial derived-ABs increase the production of an anti-apoptotic survival factor, the chemokine CXCL12, via the transfer of miR-126 to ECs. Systemic injection of miR-126-enriched ABs reduces atherosclerotic plaque size in mouse models of atherosclerosis. This atheroprotective effect is lost when mice are treated with endothelial ABs isolated from *miR-126^-/-^* mice (Zernecke et al., 2009), suggesting that this effect is mediated by the delivery of miR-126 to vascular cells. In addition to MVs, HDL and LDL particles have also been shown to contain distinct microRNAs, and the microRNAs contained in these particles are altered during atherosclerosis (Vickers et al., 2011). Interestingly, miR-223 can be transferred from HDL particles to ECs to suppress ICAM-1 expression and EC activation (Tabet et al., 2014). Taken together, these studies are beginning to shed light on the role of secreted and/or circulating microRNAs in the control of atherosclerosis. However, many aspects of the biology of secreted microRNAs in cardiovascular diseases remain unknown. For example, it is not clear whether secreted microRNAs can affect NF-κB signaling and vascular inflammation. However, the fact that circulating miR-181b levels are reduced in septic patients and in CAD patients (Sun et al., 2012, 2014), and considering the known role for miR-181b in suppressing NF-κB activity, this suggests that circulating microRNAs may indeed influence vascular inflammation. Much remains to be uncovered in this burgeoning area of research.

## RNA-BASED THERAPEUTICS TO TREAT VASCULAR INFLAMMATION

Considering the important effects that microRNAs have on controlling vascular inflammation, interest has focused on developing therapeutics that target microRNA-regulated gene expression networks. MicroRNAs are promising targets since they often regulate a network of genes in a given pathway (**Figure 1**), which may limit the development of resistance to treatment, which is a major drawback of single target therapies (van Rooij and Kauppinen, 2014). Two approaches can be envisioned: over-expressing anti-inflammatory microRNAs or inhibition of pro-inflammatory microRNAs. The over-expression of microRNAs in the vasculature can be achieved by intravascular delivery of microRNA mimics (Sun et al., 2012, 2014). This is typically achieved by liposome-mediated delivery modalities, and the effectiveness of this strategy is illustrated by studies utilizing miR-181b over-expression in ECs to inhibit vascular inflammation and atherosclerosis in mouse models (Sun et al., 2012, 2014). Phase I clinical trials are also underway to replace microRNAs that are down-regulated in liver cancer (van Rooij and Kauppinen, 2014). However, the potential for non-target cell uptake is high, and the functional effects of such off-target effects are not well understood. This is especially relevant considering the pro- and anti-atherogenic effects of NF-κB in ECs and monocytes/macrophages, respectively (Kanters et al., 2003; Gareus et al., 2008). Interestingly, systemic delivery of miR-181b inhibits NF-κB in the endothelium through the targeting of IPOA3, but because monocytes/macrophages utilize a distinct Importin for NF-κB activation, no effect on NF-κB signaling is observed in these cells (Sun et al., 2014). Additional approaches to achieve more narrow cell-specificity of ectopic microRNA expression include the use of adeno-associated viruses that have natural or engineered tropism for particular cell types (White et al., 2004; Work et al., 2006), or the use of cell-specific promoters to drive microRNA expression (Lovren et al., 2012). Recent work has also identified nanoparticles that deliver siRNAs preferentially to ECs *in vivo* (Dahlman et al., 2014). These nanoparticles may also be effective for the delivery of microRNA mimics specifically to ECs. Finally, MVs have been engineered to deliver siRNAs to specific cell types (Alvarez-Erviti et al., 2011), suggesting that this approach may also be feasible for microRNA mimic delivery to the vasculature.

Therapeutic strategies to inhibit microRNA expression have been widely used in pre-clinical studies, and are beginning to be used in clinical settings. Sustained knock-down of microRNA activity throughout the body can be achieved by intravascular or subcutaneous injection of anti-sense antagomirs, which are typically cholesterol-conjugated to improve tissue distribution (Krutzfeldt et al., 2005). These approaches are appropriate for microRNAs that are expressed exclusively in a disease condition or are expressed in a cell-restricted fashion, since it is difficult to achieve tissue-specific delivery. Such approaches have been used to inhibit miR-92a expression to promote functional recovery following ischemia/reperfusion injury in large animal (i.e., porcine) studies (Hinkel et al., 2013) or to inhibit atherogenesis in mouse models (Loyer et al., 2014). Recently, the first Phase II clinical trial for a microRNA-based therapeutic was completed, and has shown promising results. The liver-specific microRNA, miR-122, is required for Hepatitis C virus (HCV) replication (Jopling et al., 2005). Inhibition of miR-122 was previously shown to reduce HCV titer in infected chimpanzees without toxic effects or the development of resistance mutations (Lanford et al., 2010). Recent Phase II results using an anti-miR-122 drug called Miravirsen (Santaris Pharma) have revealed that five injections over the course of a month could reduce HCV viral titer in a dose-dependent fashion, and this effect was long lasting (>3 months) and did not elicit compensatory mutations in the HCV genome (Janssen et al., 2013). While some patients displayed a rise in HCV titer at later time-points following cessation of treatment, these results are very promising and suggest that similar approaches may be utilized for cardiovascular diseases. However, it is important to note that cardiovascular diseases typically develop over long periods of time, so sustained therapeutic treatment may be difficult to achieve. Acute vascular inflammatory disorders, such as sepsis and myocardial infarction may therefore be more amenable to treatment in the short term, while treatment of atherosclerosis will likely require significant advances in mimic/antagomir delivery.

## CONCLUSION

It is now abundantly clear that noncoding RNAs modulate NF-κB-driven responses during vascular inflammation. Further research will likely unearth additional noncoding RNAs, including lncRNAs, that provide additional layers of regulation to fine-tune the inflammatory response and contribute to vascular diseases such as atherosclerosis. Since aging has a large impact on the acquisition of vascular inflammation, it will be interesting to determine the effect of aging on noncoding RNA-dependent regulation of NF-κB signaling in the vasculature. The influence of circulating microRNAs on vascular inflammation will also be of significant interest. Much of the work linking microRNAs to vascular inflammation has been conducted in mouse models of acute (i.e., sepsis) or chronic vascular inflammation (i.e., atherosclerosis). Mouse models will continue to be useful in defining the role of microRNAs in vascular inflammation, by either using genetic manipulation or through the use of microRNA mimics or inhibitors. Mice and large animal models (i.e., porcine and chimpanzee) will also serve as an important pre-clinical model for the development of therapeutics aimed at microRNA-regulated pathways. Mouse models and the use of human patient samples will be highly informative for studies of the biology of circulating microRNAs. Given the limited conservation of lncRNAs, the use of model organisms may be of limited utility, and studies using cultured human cells will be necessary to understand their biology. Given the promise of microRNA-based therapeutics in Hepatitis C (Janssen et al., 2013), it will be exciting to watch for developments of therapies that harness the ability of microRNAs to regulate NF-κB signaling in vascular diseases.

## Conflict of Interest Statement

The authors declare that the research was conducted in the absence of any commercial or financial relationships that could be construed as a potential conflict of interest.
